# Retrospective real-life study on preoperative imaging for minimally invasive parathyroidectomy in primary hyperparathyroidism

**DOI:** 10.1038/s41598-022-18219-3

**Published:** 2022-10-19

**Authors:** Jacqueline Bijnens, Annick Van den Bruel, Vincent Vander Poorten, Ingeborg Goethals, Steven Van Schandevyl, Catherine Dick, Frank De Geeter

**Affiliations:** 1 Otorhinolaryngology-Head and Neck Surgery, AZ Sint-Jan, Bruges, Belgium; 2 Internal Medicine, Endocrinology, AZ Sint-Jan, Bruges, Belgium; 3 Otorhinolaryngology-Head and Neck Surgery, University Hospitals Leuven, Leuven, Belgium; 4 Department of Oncology, Section Head and Neck Oncology, Leuven Cancer Institute, KU Leuven, Leuven, Belgium; 5 Nuclear Medicine, Ghent University Hospital, Ghent, Belgium; 6 Nuclear Medicine, AZ Sint-Jan, Bruges, Belgium

**Keywords:** Endocrine system and metabolic diseases, Endocrine system and metabolic diseases, Diseases, Endocrinology, Parathyroid glands

## Abstract

The objective of this study was to retrospectively evaluate preoperative imaging modalities for localization of parathyroid adenomas with a view to enable minimally invasive parathyroidectomy and in particular, to consider the contribution of ^18^F-fluorocholine-PET/CT. 104 patients with primary hyperparathyroidism, who underwent parathyroid surgery in a single centre during a 6-year period were included. Of these, 103 underwent ultrasound, 97 ^99m^Tc-Pertechnetate/SestaMIBI-SPECT, 20 MRI and 30 ^18^F-fluorocholine-PET/CT. Based on surgical findings, sensitivities and specificities for correct lateralisation in orthotopic locations were: for ultrasound 0.75 (0.65–0.83) and 0.89 (0.81–0.94), for ^99m^Tc-MIBI-SPECT 0.57 (0.46–0.67) and 0.97 (0.91–0.99), for MRI 0.60 (0.36–0.81) and 0.83 (0.59–0.96) and for ^18^F-fluorocholine-PET/CT 0.90 (0.73–0.98) and 0.90 (0.73–0.98). Correctly lateralized adenomas were significantly larger than those not found with ultrasound (p = 0.03) and SPECT (p = 0.002). Pre-operative PTH-levels were higher in single adenomas detected by scintigraphy than in those not (p = 0.02). 64 patients could be treated with a minimally invasive procedure. Cure after parathyroidectomy was obtained in 94% of patients. ^18^F-Fluorocholine-PET/CT could be shown to be a highly accurate modality to localize parathyroid adenomas preoperatively, obviating the need for total exploration in the majority of patients in whom ultrasound and scintigraphic results are discordant or both negative.

## Introduction

### Background

Primary hyperparathyroidism (pHPT) is a common condition with an incidence of 66 women and 25 men/100,000 per year^1^. Classically the condition is characterised by hypercalcemia along with elevated or inadequately normal PTH concentrations in the blood. Normocalcemic pHPT is defined by normocalcemia and elevated PTH levels^2^. Possible complications include renal lithiasis, depressive mood and osteoporotic bone fractures. pHPT is most frequently caused by a single adenoma, sometimes by multiple adenomas or hyperplasia and rarely by carcinoma. In some cases watchful waiting or medical treatment is proposed but surgery with removal of the causing abnormal parathyroid is the only definitive treatment^2,3^. Traditionally, due to lack of specific imaging, bilateral neck exploration with inspection of all 4 parathyroids had to be performed, to identify and remove the one(s) which appeared abnormal. Nowadays a minimally invasive approach is the preferrable approach in localized single adenomas. Compared with neck exploration minimally invasive parathyroidectomy (MIP) results in shorter operative times, lower complication rates (especially hypoparathyroidism and recurrent laryngeal nerve injury), less scarring and less morbidity^4,5^. The current standard is a combination of ultrasonography (US) and ^99m^Tc-Pertechnetate/SestaMIBI-SPECT, further shortened as ^99m^Tc-MIBI-SPECT, for preoperative localization of the adenoma(s). It is preferable to have 2 matching imaging modalities for planning a MIP^4,5^. However, when these two ‘first-line’ investigations are negative or inconsistent, an indication arises for additional localizing techniques such as MRI (Magnetic Resonance Imaging), 4D-CT or ^18^F-fluorocholine-PET/CT, further shortened as FCh-PET/CT, with the latter gaining popularity in the last few years. FCh-PET/CT was developed for staging and follow-up of prostate carcinoma, and was first reported to incidentally show a parathyroid adenoma in 2013^6^. In parathyroid adenomas, upregulation of phospholipid calcium/AMPc-dependent choline kinases is triggered by higher levels of circulating PTH, and thereby facilitates uptake of Fluorocholine which is a phospholipid analogue^7^.

The purpose of the present study was to evaluate the preoperative imaging modalities for localization of parathyroid adenomas and in particular to consider the contribution of FCh-PET/CT.

## Methods

### Patients

In this retrospective study, patients who underwent parathyroid surgery over a 6-year time period from 01-01-2014 to 31-12-2019 in AZ Sint-Jan, Bruges, Belgium were identified. Patients with a diagnosis of pHPT with one or multiple adenomas were included. Exclusion criteria were secondary/tertiary hyperparathyroidism (n = 54), concurrent head-neck malignancies (n = 4), MEN syndrome (n = 3) and missing patient record (n = 3). A total of 104 patients were included. Patient records were reviewed. Information about the preoperative investigations that were used for diagnosis and surgical planning, type of surgery and histology were collected from each file. Other data extracted included descriptive parameters, pre- and postoperative blood results.

### Preoperative imaging modalities

Patients in our study cohort were referred for parathyroidectomy by three on-site and four external endocrinologists. Localization of the parathyroid adenoma was preoperatively assessed using neck ultrasound, ^99m^Tc-MIBI-SPECT, and in some cases MRI, FCh-PET/CT and/or 4D-CT, according to clinical need and availability. Most ultrasound studies were performed by one of our centre’s experienced endocrinologists using Aixplorer Supersonic Image (Aix-en-Provence, France). Parathyroid scintigraphy was performed in our centre for the majority of patients, using a dual tracer (111 MBq ^99m^Tc-pertechnetate, 740 MBq ^99m^Tc-SestaMIBI) technique with SPECT/CT on a SymbiaT16 (Siemens, Erlangen, Germany). The effective dose for the SPECT part is estimated at 8.1 mSv. Total time for this investigation is approximately 90 min. In four patients evaluated elsewhere no hybrid SPECT/CT was performed. MRI with gadolinium contrast took place at our hospital with 3 T MRI devices (Ingenia 3.0 T or Ingenia Elition 3.0 T, Philips, Eindhoven, the Netherlands). FCh-PET/CT was performed at the University Hospital Ghent on a Siemens Biograph mCT 20 Flow PET/CT scanner (Siemens, Erlangen, Germany) with F-18-labelled fluoro-methyl-choline as a tracer, at a dose of 5 MBq/kg. Scanning was done from head to diaphragm to cover possible ectopic localizations. The effective dose for the PET part is estimated at 6.1 mSv. FCh-PET/CT takes approximately 30 min from tracer injection till the end of the scan.

From the reports of these imaging studies, all by experienced imaging specialists, the resulting localization of the parathyroid adenomas was classified as left, right, bilateral, none or ectopic. Given the limited implications for operative proceedings, we did not address the upper versus lower quadrant allocation in the analysis presented here. An imaging result was regarded as correct if it identified either the correct lateralization (for orthotopic localizations) or the correct ectopic localization of the parathyroid adenoma(s). Two imaging modalities pointing at different locations were labelled as discordant.

### Surgery, histopathology & follow-up

Parathyroidectomy was performed by one of two experienced ENT head and neck surgeons (C.D. and T.V.). If the adenoma was localised preoperatively, surgery was performed through an incision of 2.5–3 cm or less. In the event of not finding the parathyroid adenoma in the designated area, the minimal invasive surgery was converted to a neck exploration. When there was no preoperative localization, a bilateral neck exploration was planned. Definite localization (originating from parathyroid a, b, c or d) was determined peroperatively and this was confirmed with peroperative PTH-level monitoring and frozen section. The adenoma size used for our analyses was taken from the pathology report as the largest diameter of the specimen, when available (86 patients). Surgical findings with histopathologic confirmation of hyperfunctioning (hyperplastic or adenomatous) parathyroids were used as the standard against which to judge the imaging techniques. Patients were followed up by their referring endocrinologist. Cure was defined as normocalcemia 6 months after surgery^2^.

### Statistical methods

All statistical analyses and graphics were performed in R version 4.0.1 and figures were produced using the package ggplot2. Sensitivity and specificity of imaging modalities for lateralization were calculated in all patients in whom they were available, with 95% confidence intervals (CI) according to Clopper-Pearson. To avoid patient selection bias, sensitivity and specificity were compared pairwise between tests available in the same patients, using the exact McNemar test. The Mann–Whitney U test and Fisher exact test were used to compare continuous and categorical variables, respectively. P-values less than 0.05 were considered significant.

### Ethical considerations

This retrospective study was approved by the local Ethics Committee of AZ Sint-Jan Brugge-Oostende (Approval number 2579). All research was performed in accordance with relevant guidelines.

### Informed consent

Our manuscript does not relate an experiment, but merely a retrospective chart review of data available. In no way did this retrospective study interfere with patient’s diagnostic workout, treatment or follow-up. Therefore, informed consent was waived by the local ethical committee: Commissie voor Ethiek AZ Sint-Jan Brugge-Oostende av.

## Results

In two patients out of 104 included, no definitive adenoma localization was found. One-hundred-and-seven adenomas were found in the remaining 102 patients; 5 patients had 2 adenomas. The study cohort consisted of 25% men (n = 26) and 75% women (n = 78). The average age at the time of parathyroidectomy was 58 ± 12.67 years. Mean preoperative serum PTH-level was 137.57 ng/L, ranging from 24 to 952 ng/L. Mean preoperative corrected serum calcium was 2.81 mmol/L (SD ± 0.22). Main size of the diseased parathyroids was 17.9 ± 9.4 mm. A summary of descriptive statistics is contained in Table 1.

**Table 1:** Overview of basic characteristics.

Patients	104
**Adenomas**^**a**^	107
Single	95
Double	6
Size (mm) (n = 91)^b^	18 (± 9.6)
**Gender (n = 104)**	
Male	25% (n = 26)
Female	75% (n = 78)
Age, (years)^b^	58 (± 13)
Preoperative serum PTH, ng/L^c^	138 (24–952)
Preoperative serum corrected calcium mmol/L^b^	2.81 (± 0.22)
**Surgical procedure**	104
Minimal invasive parathyroidectomy	68 (65%)
Classic bilateral four gland exploration	36 (35%)

^a^In 2 patients no definitive adenoma localization was confirmed.

^b^Mean ± standard deviation.

^c^Mean (range).

Distribution of the parathyroid adenomas based on surgical localization was as follows: 17 originating from upper left parathyroid (a), 32 from lower left (b), 31 from lower right (c) and 25 from upper right (d).

Two adenomas were situated ectopically, one posterior to the oesophagus and one retrosternally. This means 59% of parathyroid adenomas originated from one of the lower parathyroids.

Sixty-eight patients had a MIP and 36 patients had a bilateral neck exploration. In five patients bilateral exploration followed peroperative conversion. In 31 patients a bilateral exploration had been planned for the following reasons: negative or discordant pre-operative localization in 23 patients (in 2 of these thyroid surgery was planned as well), synchronous thyroid disease requiring surgery in another five patients, suspicion of parathyroid carcinoma in two and presumed bilateral adenomas in one patient. Five patients had previous neck surgery, of whom 3 could be treated with MIP. Overnight stay in the hospital was 1 or 2 days in 96 patients (92%). One patient had a wound infection and persistent hypocalcemia occurred in another patient. Cure rate was 94% in our patient group, which also included double adenomas, surgery combined with thyroid surgery and patients in which a definite adenoma localisation was not identified. At the closure of the database, four patients had undergone repeat surgery.

Table 2 details imaging results and their relationships in our study population. When ultrasound and scintigraphy were concordant, they always were correct and none of these patients needed conversion to a complete exploration.

**Table 2:** Imaging results according to surgical findings and according to concordance or discordance of ultrasound and scintigraphy.

Surgical result	n	Concordance of Ultrasound & ^99m^Tc-MIBI-SPECT	N	Ultrasound		^99m^Tc-MIBI-SPECT		Fch-PET/CT		MRI	
				Correct	Incorrect	Correct	Incorrect	Correct	Incorrect	Correct	Incorrect
Orthotopic unilateral adenoma	96	Concordant	37	*37*		*37*		*4*	**1 (extra)**	*3*	**1 (missed)**
		Not concordant	6	*3*			**2 (extra)** **1 (contralat.)**				**1 (contralat.)**
					**1 (extra)** **2 (contralat.)**	*3*					**1 (missed)**
		Only suggestion on US	27	*21*			**21 (missed)**	*12*	**2 (contralat.)**	*2*	
					**2 (extra)** **4 (contralat.)**		**2 (missed)** **4 (missed)**				**2 (contralat.)**
		Only suggestion on ^99m^Tc-MIBI-SPECT	7		**7 (missed)**	*7*				*2*	**1 (missed)**
		Both techniques negative	11		**11 (missed)**		**11 (missed)**	*1* *3*	**1 (missed)**	*1* *1*	**1 (missed)** **1 (missed)**
		Not both performed	8								
		Only ultrasound		*7*				*4*			
		Only ^99m^Tc-MIBI-SPECT				*1*				*1*	
Ectopic adenoma	2	Concordant	1	*1*		*1*					
		Not concordant	1		**1 (Missed ectopic, 2 extra orthotopic)**		**1 (missed ectopic)**	*1*			
Orthotopic bilateral adenoma	4	Concordant	1	*1*		*1*					
		Not concordant	2	*1*			**1 (missed 1 location)**	*1*		*1*	
					**1 (missed 1 location)**	*1*					
		Only suggestion on US, ^99m^Tc-MIBI-SPECT negative	1		**1 (missed 1 location)**		**1 (missed 2 locations)**				
No parathyroid adenoma found	2	Not concordant	2		**2 (extra)**	*2*					**1 (extra)**
Total	104		104	103		97		30		20	

Data per patient (not per adenoma) was sorted by definitive localization (ie. the surgical result), followed by concordance of the conventional imaging modalities. ‘Concordant’ means both ultrasound and 99mTc-MIBI-SPECT pointed out the same localization. If they suggested different sites of adenoma localization, this was labelled ‘Not concordant’. When one or both of these imaging modalities was not showing any parathyroid adenoma, this was labelled as ‘Only suggestion on US, 99mTc-MIBI-SPECT negative’, ‘Only suggestion on 99mTc-MIBI-SPECT, ultrasound negative’ and ‘Both techniques negative’. When ultrasound and 99mTc-MIBI-SPECT were not both performed this was also indicated. Further findings are specified in the italics font (pre-operative correct suggestion of localization) and bold columns (pre-operative incorrect suggestion of localization). When the imaging results did not match the eventual localization result, the nature of the mistake was specified:—contralateral suggestion of localization of the adenoma (contralateral),—negative result on imaging (missed),—showed the correct localization but also an extra localization on the contralateral side, or in the case of no definitive localization, did show localizations on the imaging without showing any other localizations (extra). Per row, from left to right, the results of the imaging modalities for the same patients can be compared.

For the detection of orthotopic adenomas in the right or left bed, sensitivity for ultrasound (n = 101 patients evaluable) was 0.75 (CI 0.65–0.83) and specificity 0.89 (CI 0.81–0.94). For ^99m^Tc-MIBI-SPECT (n = 95) sensitivity was 0.57 (CI 0.46–0.67) and specificity 0.99 (CI 0.94–1.00). For MRI (n = 19) we found a sensitivity of 0.60 (CI 0.36–0.81) and specificity of 0.83 (CI 0.59–0.96). FCh-PET/CT (n = 30) showed a sensitivity of 0.90 (CI 0.73–0.98) and specificity of 0.90 (CI 0.73–0.98). In a head-to-head comparison between imaging modalities used in the same patient, ultrasound had significantly higher sensitivity than ^99m^Tc-MIBI-SPECT (p = 0.006), but ^99m^Tc-MIBI-SPECT had higher specificity than ultrasound (p = 0.03), resulting in similar accuracy. FCh-PET/CT was significantly more sensitive (p = 4E−06) and accurate (p = 0.001) than ^99m^Tc-MIBI-SPECT, for similar specificity.

Both ultrasound and ^99m^Tc-MIBI-SPECT correctly picked up one of two ectopic adenomas; FCh-PET/CT was only performed in the other ectopic adenoma and showed it correctly.

**Figure 1:**
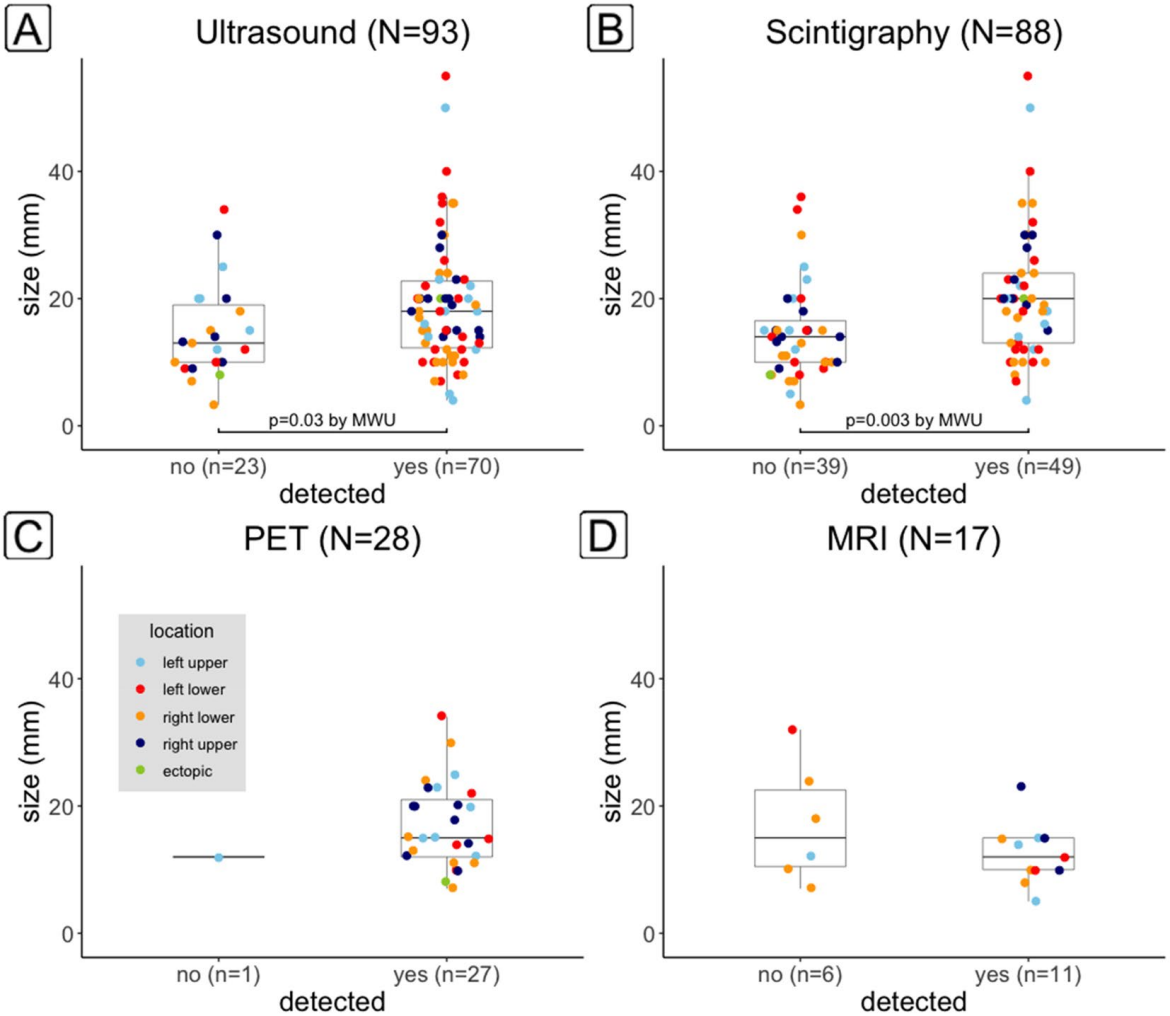
Adenoma size according to whether they were detected in the correct (left, right, ectopic) location or not by ultrasound (**A**), scintigraphy (**B**), 18FCh-PET/CT (**C**) or MRI (**D**). Only lesions whose size was mentioned in the histology report were included. For ultrasound and scintigraphy, adenomas detected were on average larger than those not detected. Location of the adenomas was colour coded.

Adenomas correctly found on ultrasound were significantly larger than those missed (p = 0.03) (Fig. 1).

For ^99m^Tc-MIBI-SPECT, a similar difference was even more significant (p = 0.002). For FCh-PET/CT no meaningful comparison was possible since all but one adenoma evaluable were lateralized correctly. For MRI, no significant difference was found. The smallest adenoma detected both with ultrasound and with ^99m^Tc-MIBI-SPECT measured 4 mm, with FCh-PET/CT 7 mm and with MRI 5 mm. The largest adenomas missed measured 34 mm for ultrasound, 36 mm for ^99m^Tc-MIBI-SPECT, 12 mm for FCh-PET/CT and 32 mm for MRI. Only lesions whose size was mentioned in the histology report were included for this analysis.

**Figure 2:**
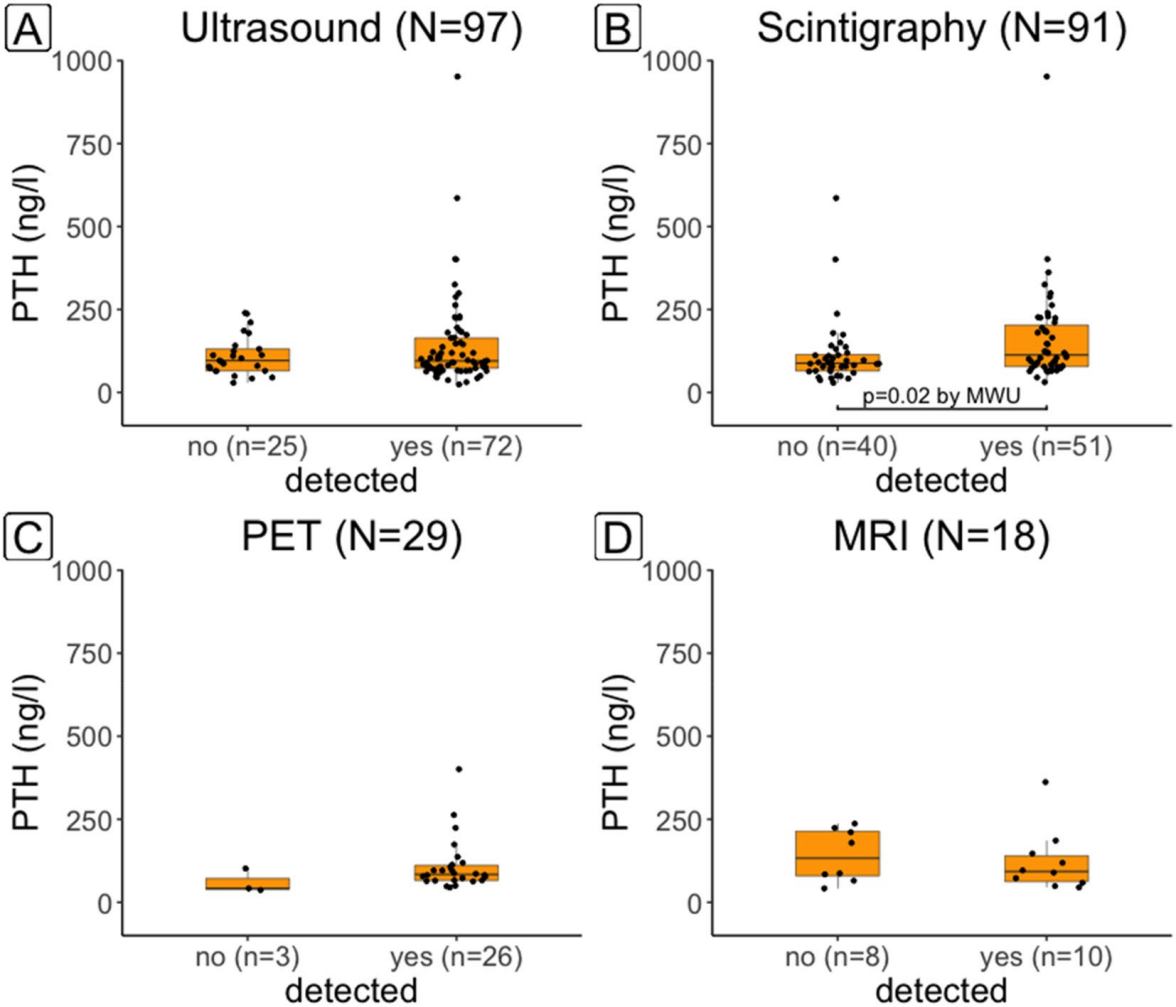
Preoperative serum PTH according to whether adenoma was detected in the correct (left, right, ectopic) location or not by ultrasound (**A**), scintigraphy (**B**), 18FCh-PET/CT (**C**) or MRI (**D**). Only single adenomas were used for this analysis. For scintigraphy, PTH was on average higher when the adenoma was detected than when it was not.

In patients with single adenomas, pre-operative PTH-levels were higher when the adenoma was detected by ^99m^Tc-MIBI-SPECT than when it was not (p = 0.02) (Fig. 2).

For ultrasound and MRI, no significant difference was found. For FCh-PET/CT no meaningful comparison could be made due to small sample size.

FCh-PET/CT was more often performed when ultrasound and ^99m^Tc-MIBI-SPECT results were discordant than when these agreed (p = 0.03). Nine out of 14 FCh-PET/CT’s performed in patients with discordant ultrasound and ^99m^Tc-MIBI-SPECT results obviated total explorations. Likewise, 2 out of 3 FCh-PET/CT studies performed in patients with both negative ultrasound and ^99m^Tc-MIBI-SPECT, and all 3 FCh-PET/CT studies in patients in whom ^99m^Tc-MIBI-SPECT had not been performed, enabled MIP.

## Discussion

Preoperative localization of adenomatous parathyroid glands in patients with pHPT is essential for planning MIP. We report a retrospective cohort study, evaluating ultrasound, ^99m^Tc-MIBI-SPECT, MRI and FCh-PET/CT for their diagnostic performance in this setting.

In our study, the majority (59%) of adenomas originated from one of the lower parathyroids, which is congruent with the findings in a large series by LoPinto et al. (n = 810) and Reid et al. (n = 199), stating that lower parathyroids are more often affected in primary hyperparathyroidism^3,8^.

In our series, ultrasound had a sensitivity of 75% and specificity of 89% for allocating the adenomatous parathyroid to the right or left thyroid bed. ^99m^Tc-MIBI-SPECT had a sensitivity of 57% and specificity of 99%. These figures are consistent with reported ranges in the literature^9–13^. MRI showed a sensitivity of 60% and specificity of 83%, also in line with other reports^14^. FCh-PET/CT demonstrated a sensitivity and specificity of both 90%. Similar figures have also been reported by several other authors, with per-lesion based sensitivities around 80–96%^7,15^. Since patients have to be referred to another institution for FCh-PET/CT, which is not available in-house, this technique was more often turned to when primary imaging was discordant. In a head-to-head comparison in this selected population, FCh-PET/CT was more sensitive and accurate than ^99m^Tc-MIBI-SPECT, for similar (high) specificity.

Not only do the high detection rate and low rate of false positives make FCh-PET/CT a promising imaging modality in treatment planning for parathyroid surgery in primary pHPT, it has two additional advantages. Firstly, radiation burden to the patient is less than with other scintigraphic techniques^10^. The exact radiation dose of course is strongly dependent on the details of the imaging protocol, including the injected dose of the tracer(s) and the settings for the CT component, but the shorter half-life of the PET tracer always results in lower doses. Secondly, the duration of FCh-PET/CT is considerably shorter making it more patient friendly, approximately 30 min for FCh-PET/CT versus 90 min for ^99m^Tc-MIBI-SPECT. If more readily available, FCh-PET/CT may replace ^99m^Tc-MIBI-SPECT as a first-line investigation.

Adenomas correctly lateralized on ultrasound or ^99m^Tc-MIBI-SPECT on average were larger than those missed (Fig. 1). A similar size effect has been described previously^16,17^. For FCh-PET/CT, which localized all but 1 adenoma with reported size correctly, no meaningful comparison was possible in our cohort, but studies with larger sample sizes found the effect of size on detection rates to be less significant for FCh-PET/CT compared with conventional imaging. This differential size effect can be accounted for by the better spatial resolution of PET compared with SPECT^18^. For MRI, we did not find a significant size effect either; correctly lateralized adenomas were on average even smaller than those not found.

Alharbi (n = 42) reported a correlation between serum PTH-level and uptake of ^18^F-fluorocholine in parathyroid adenomas^19^. Our series (n = 29) comprised only 3 single adenomas that were not detected; these tended to have lower PTH than the single adenomas that were detected, but the difference was not significant in this small group; this was similar to the findings of Grimaldi in 21 patients and Kluijfhout in 44 patients^20,21^. On the other hand, we found that PTH was on average significantly higher in adenomas detected than in those missed by ^99m^Tc-MIBI-SPECT, confirming the findings of Mihai and Khorasani^16,22^.

Limitations of the present study firstly include its retrospective design and relatively small sample size. However, the population included was homogeneous with respect to the exclusion of secondary/tertiary hyperparathyroidism and MEN syndrome. Also the cure rate described (94%) is in accordance with other reports^3,23,24^. Secondly, because of referrals from different centres, first line preoperative imaging varied in the modalities used and in their technical details. While this precluded large-scale head-to-head comparisons, it may well represent the real-life situation. Lastly, since the study cohort comprises surgical patients only, patients with mild, asymptomatic primary hyperparathyroidism and absence of a stringent surgical indication are not represented in this study cohort. The selection of surgical patients may therefore overestimate the sensitivity of the preoperative imaging techniques. Nevertheless, the selection of a surgical cohort allows for the use of a surgically and histologically confirmed parathyroid adenoma as the ‘gold standard’.

In conclusion, due to its high sensitivity and specificity, ^18^F- fluorocholine-PET/CT can be of great value for localizing parathyroid adenomas in patients with pHPT, even when ultrasound and ^99m^Tc-MIBI-SPECT are negative or discordant. The advantages of FCh-PET/CT are a high spatial resolution, a short scanning time and a relatively low radiation dose. It is therefore conceivable that FCh-PET/CT will supplant conventional ^99m^Tc-MIBI-SPECT techniques in hospitals in which it is available.

## Data Availability

The datasets generated analysed during the current study are available from the corresponding author on reasonable request.
